# Grapevine Virology in the Third-Generation Sequencing Era: From Virus Detection to Viral Epitranscriptomics

**DOI:** 10.3390/plants10112355

**Published:** 2021-10-31

**Authors:** Vahid Jalali Javaran, Peter Moffett, Pierre Lemoyne, Dong Xu, Charith Raj Adkar-Purushothama, Mamadou Lamine Fall

**Affiliations:** 1 Saint-Jean-sur-Richelieu Research and Development Centre, Agriculture and Agri-Food Canada, Saint-Jean-sur-Richelieu, QC J3B 3E6, Canada; vahid.jalalijavaran@agr.gc.ca (V.J.J.); pierre.lemoyne@canada.ca (P.L.); dong.xu@canada.ca (D.X.); 2 Département de Biologie, Centre SÈVE, Université de Sherbrooke, Sherbrooke, QC J1K 2R1, Canada; Peter.Moffett@USherbrooke.ca; 3 Département de Biochimie, Faculté de Médecine des Sciences de la Santé, 3201 rue Jean-Mignault, Sherbrooke, QC J1E 4K8, Canada; charith.raj.adkar.purushothama@usherbrooke.ca

**Keywords:** grapevine, viral disease, diagnostic methods, RNA sequencing, nanopore sequencing technology, RNA modifications

## Abstract

Among all economically important plant species in the world, grapevine (*Vitis vinifera* L.) is the most cultivated fruit plant. It has a significant impact on the economies of many countries through wine and fresh and dried fruit production. In recent years, the grape and wine industry has been facing outbreaks of known and emerging viral diseases across the world. Although high-throughput sequencing (HTS) has been used extensively in grapevine virology, the application and potential of third-generation sequencing have not been explored in understanding grapevine viruses and their impact on the grapevine. Nanopore sequencing, a third-generation technology, can be used for the direct sequencing of both RNA and DNA with minimal infrastructure. Compared to other HTS methods, the MinION nanopore platform is faster and more cost-effective and allows for long-read sequencing. Due to the size of the MinION device, it can be easily carried for field viral disease surveillance. This review article discusses grapevine viruses, the principle of third-generation sequencing platforms, and the application of nanopore sequencing technology in grapevine virus detection, virus–plant interactions, as well as the characterization of viral RNA modifications.

## 1. Introduction 

In 2020, over 7.3 million hectares of agricultural land worldwide were planted with grapevines. The economies of many countries are influenced by wine, table grape, raisin, seed oil, alcoholic beverage, and vinegar production [1], as well as associated tourism activities. At present, most cultivated grapevines belong to varieties of *V. vinifera* from Eurasia [2,3]. Climate change and the global movement of plant material on a large scale have led to the emergence of new viral diseases, which has become a serious concern in plant production. Indeed, the global negative economic impact of viral diseases on plant products has been estimated at USD 30 billion annually in 2014 [4]. The economic impact of viral infections in grapevine production is considerable. For instance, grapevine leafroll disease (GLRD) can have an adverse economic effect of between USD 29,902 and USD 226,405 per hectare [5]. In addition, the average yield loss during GLRD outbreaks in a vineyard can vary between 15% and 20% and is sometimes as high as 40% [6].

More than 80 viruses from 17 families and 34 genera can infect grapevine (Table S1). Approximately half of these viruses result in viral diseases that can be classified into four major classes: rugose wood complex, leafroll, leaf degeneration, and fleck disease. In recent years, new viral species, such as *Grapevine red blotch virus* and *Grapevine Pinot gris virus*, as well as new strains of known viruses, have been identified and detected through high-throughput sequencing (HTS) technologies. These viruses cause substantial negative impacts on grapevine production by reducing vine growth, fruit production, and fruit quality [7,8,9,10]. 

Early identification of viruses allows growers to take quick actions and effective sanitary measures, such as removing infected vines, limiting the movement of agricultural machinery, cleaning tools, controlling vectors to limit the spread of viruses, and providing improved tools for certification procedures of propagative materials before importing germplasm [9,11]. Some common diagnostic tools such as serological [12,13], nucleic acid amplification-based [14,15], microarrays [16,17], and multispectral [18] or hyperspectral [19,20] imaging methods have been used for monitoring and detecting grapevine viruses. 

With the advent of HTS, the characterization and investigation of known and novel viruses have been made possible. Although many viruses have been detected and identified by total RNA extraction and sequencing, the abundance of viral sequences relative to cellular RNAs is often very low, and the detection of low-titer viruses may be compromised. Some extractionmethods have been developed to concentrate viral nucleic acids, thus improving the abundance of viral sequences submitted to HTS. These methods include virion-associated nucleic acids (VANAs) and double-stranded RNA (dsRNA) extraction, small interfering RNA (siRNA), and rRNA depleted and polyadenylated RNA isolation. Many known and novel grapevine viruses have been detected and identified with these methods, such as *Grapevine Syrah virus 1* (GSyV-1), through total RNA and dsRNA sequencing, *Grapevine vein-clearing virus* (GVCV) and *Grapevine Pinot gris virus* (GPGV), through small RNA sequencing, *Grapevine virus F* (GVF) and *Grapevine red blotch virus* (GRBaV), through dsRNA sequencing, and *Grapevine Roditis leaf discoloration-associated virus* (GRLDaV), through siRNA sequencing [21,22,23,24,25]. Although second-generation sequencing has been broadly used in virus identification and detection, several limitations remain, such as read lengths, GC content and amplification biases, laborious and costly library preparation methods, data management, and the requirement of sophisticated technical expertise for data analysis [26,27].

There are many unanswered questions about the biological and epidemiological features of individual grapevine viruses in mixed infections, the molecular basis of plant–virus interactions, and grapevine virome complexity. Therefore, in this review, we briefly introduce the principle of third-generation sequencing platforms and explain the application of nanopore technology in several areas of research in grapevine virology.

## 2. Third-Generation Sequencing Platforms 

Through the advent of third-generation sequencing technologies, such as PacBio single-molecule real-time (SMRT) sequencing and Oxford Nanopore sequencing, some of the aforementioned limitations of second-generation sequencing have been addressed. In SMRT sequencing, a double-stranded DNA template is circularized, called SMRTbell, through the ligation of hairpin adapters to both ends. The template and DNA polymerase are immobilized at the bottom of a chip, named the SMRT Cell, consisting of many photonic nanostructures, zero-mode waveguides (ZMWs). The latest generation of SMRT sequencer has one million ZMWs per SMRT Cell. Single-molecule real-time sequencing does not require an amplification step, and the template is sequenced based on its complementary strand synthesis by DNA polymerase with fluorescently labeled dNTPs [28]. After loading a SMRTbell library into a SMRT Cell, the DNA polymerase starts replicating the DNA from the adapter region of the SMRTbell using four fluorescently labeled A, T, G, and C nucleotides. Each nucleotide produces a signature light pulse that is captured as a “movie”. Light pulses are then basecalled to the nucleotide sequence, and each sequence obtained from a ZMW is referred to as a Continuous Long Read (CLR) [29]. The Sequel II SMRT sequencer can produce reads as long as 50 kb. Although the library preparation and sequencing running time of SMRT sequencing are shorter than short-read sequencing technologies, SMRT reads typically have a high error rate (~15%) [30]. However, PacBio has released a new sequencing system, circular consensus sequencing (CCS), which produces high-fidelity reads (HiFi) with 99.8% accuracy and an average length of 13.5 kb. In this system, since the DNA template is circular, the polymerase can start to synthesize several subreads from a DNA template continuously, and a single-molecule circular consensus sequence (known as HiFi) with high accuracy can be achieved [31]. 

**Figure 1:**
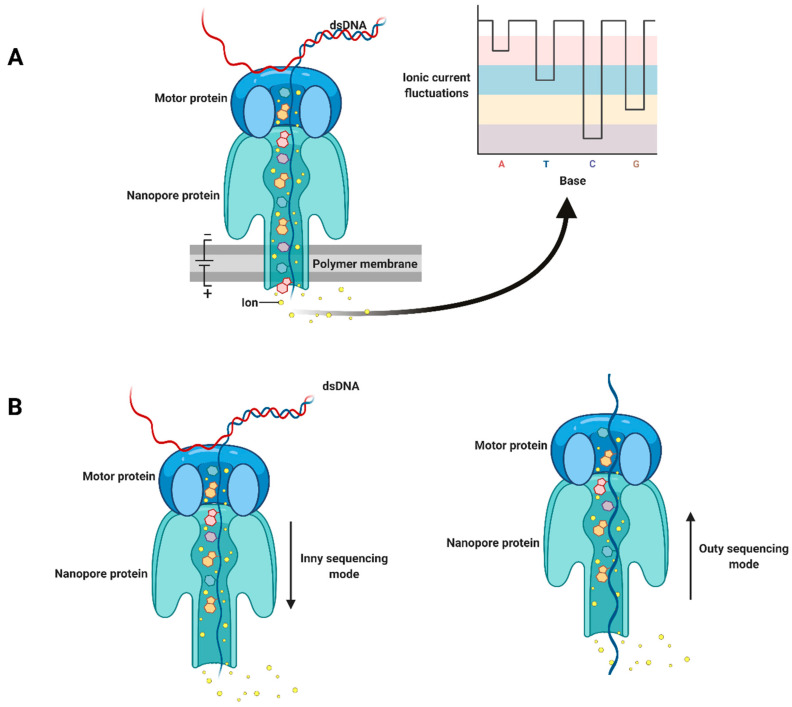
Schematic diagram of nanopore sequencing technology. (**A**). Double-stranded DNA is unwound by a motor protein. One strand of DNA is passed through a nanopore protein that forms a channel in an artificial membrane to which an ionic current has been applied. The ion current is altered depending on the identity of the base passing through the channel. The resulting fluctuations are decoded and translated to DNA (or RNA) sequence. (**B**). Inny and outy sequencing modes. Adapted from “Nanopore Sequencing”, by BioRender.com (2021). Retrieved from https://app.biorender.com/biorender-templates (accessed 15 September 2021).

In 2014, a pocket-sized commercial sequencer device, MinION, was introduced by Oxford Nanopore Technologies (ONT). The concept of nanopore sequencing was initially described in 1996 [32]. Nanopore technology is based on directly detecting each base of a single strand of nucleotides (DNA or RNA) as it passes through a nanopore protein, MspA, which is stabilized in an electrically resistant polymer membrane. The passage of single strands of nucleotides through an MspA nanopore is mediated by a helicase motor protein that unwinds double-stranded DNA and controls the translocation speed. At the same time, an ionic current is passed across the membrane, and when the nucleotides cross the nanopore, a biosensor records the variations in ionic current. The presence of each of the four possible different bases induces a specific fluctuation pattern in the ionic current, and a basecaller software can then translate these fluctuations in current to sequence information (Figure 1A) [33]. MinKNOW, as the operating software, controls nanopore sequencing devices and performs several tasks, such as data acquisition, real-time analysis and feedback, local basecalling, and data streaming. In addition, MinKNOW has several options to adjust run parameters, sample identification, and tracking, and it checks that the platform chemistry is working correctly to run the samples. 

Nanopore sequencing technology has lower throughput and high error rates compared with second-generation sequencing technologies. However, several features, such as the small size of the sequencer, ease of library preparation, low sequencing cost, and short run times, make it an interesting tool for the surveillance of viruses and other pathogens [34]. To date, two different nanopore chemistries, R9 and R10, which use nanopore proteins with properties conducive to different applications, have been used for designing nanopore flow cells. In nanopore sequencing, RNA/DNA libraries are loaded onto flow cells, which are composed of three parts: sensing chemistry (R9 and R10), a sensor array (containing the nanopores), and electronics (application-specific integrated circuit (ASIC), heat mat, and connector pins). There are three different flow cells (MinION, PromethION, and Flongle), which differ in sequencing throughput and number of nanopores (Table 1). A MinION flow cell possess 512 channels; each channel contains four nanopores, which are stabilized within four microwells. PromethION and Flongle flow cells have 2675 and 128 channels, respectively, which can be used for large and small genomes or genes, respectively. These combinations of flow cell types and sequencer machines allow for flexibility in choosing the most appropriate strategy for sequencing with respect to choosing, for example, high coverage of single samples versus multiplexing. These platforms have sequencing accuracy rates between 97% and 98.3%, depending on the flow cell chemistry and the mode of basecalling step. 

In recent years, ONT has improved the read quality of nanopore sequencing by changing the chemical reagents used in library preparation kits and flow cells and developing improved algorithms for basecalling. For example, higher raw read accuracy (98.3%) was achieved by a new basecaller software, Bonito [35]. Additional improvements include sequencing kits based on a new chemistry, Q20+, which has been tested in nanopore flow cells and will be released in the near future. This technology uses a refined motor protein (E8.1), which increases the raw read accuracy to 99.3%. Other experiments are being tested to improve nanopore sequencing. For example, selective sequencing will soon be possible, which means that by changing the DNA orientation sequencing, the length of each fragment can be measured by a nanopore. Then, the shorter fragment is ejected from the nanopore, and only desirable fragments can be sequenced. In the current sequencing mechanism, the DNA strand is passed and sequenced through the nanopore from the top environment of the nanopore to the area underneath, referred to as an “Inny” orientation. In contrast, in the Outy orientation, first, the DNA strand is passed through the nanopore, and its length is measured. The sequencing step is next performed by reversing the movement of the DNA from the inner to the top environment of the nanopore (Figure 1B).

## 3. The Application of Nanopore Sequencing to Plant Virus Detection

The potential of nanopore sequencing for the detection and identification of plant pathogens in field and lab environments has been shown in many different studies. Different library preparation kits with straightforward protocols are available and have been used to detect a number of different viruses from infected plant samples (Table 2). For instance, a field diagnosis system based on nanopore sequencing has been developed to rapidly (3 hours) detect Cassava mosaic begomoviruses in cassava plants [36]. In another study, a comparison between RNA and PCR-cDNA nanopore sequencing kits for *plum pox virus* detection in tobacco samples indicated that nanopore technology could effectively identify plant viruses [34]. However, the results of the latter study showed that the PCR-cDNA sequencing kit could produce longer reads and 400-times more reads than the direct RNA sequencing kit. In addition, the mean quality score and mean read length were higher for the PCR-cDNA sequencing kit compared to the direct RNA sequencing kit [34]. Therefore, based on the goals of the diagnostic or experimental approach, the choice of sequencing kit requires careful consideration.

The positive features of nanopore sequencing, such as quick library preparation protocols, pocket-sized sequencer machines, producing many sequences in few hours, and being a cost- and time-effective method, have the potential to dramatically impact plant pathology, especially in the area of diagnostics. In particular, this technology can be used for the parallel sequencing of multiple samples through multiplex barcoding. Our research group at Agriculture and Agri-Food Canada’s Saint-Jean-sur-Richelieu Research and Development Centre has been working on optimizing this technology to detect grapevine viruses. Preliminary results indicate that not only is this technology cheaper than HTS but also the diagnostic capabilities are similar to Illumina (unpublished data) and could be an attractive alternative diagnostic tool for grapevine viruses.

Below, we discuss different library preparation protocols that can be used for the detection and identification of grapevine viruses. In addition, we describe how nanopore sequencing can be used for the identification of different long non-coding RNAs and circular RNAs involved in grapevine virology, as well as its potential to study plant or viral RNA modification. 

## 4. Detection of Grapevine RNA Viruses by Nanopore Direct cDNA and RNA Sequencing

Grapevine viruses are represented by many families, including viruses with RNA or DNA genomes [9]. Different types of material, such as total RNA or DNA, double-strand RNAs (dsRNAs), and small interfering RNAs (siRNA), can be targeted for sequencing the viruses infecting grapevine. Due to the presence of plant RNAs and rRNAs, detecting low-titer viruses is challenging when using total RNA extraction for sequencing and is also an important consideration if one wishes to reduce the sequencing complexity to allow for multiplexing samples [44]. An alternative option for virus sequencing is to use dsRNA. Plant RNA viruses produce dsRNA as an intermediate during their replication. DNA viruses have also been shown to produce dsRNA during infection, possibly due to the action of endogenous RNA-dependent RNA polymerases [45,46]. Therefore, dsRNA sequencing increases the overall proportion of viral reads and detection sensitivity [44]. Using this method, we have recently characterized the virome of some grapevine cultivars in several Quebec vineyards, and many viruses were detected by Illumina sequencing [22]. However, dsRNA extraction is time-consuming compared to total RNA extraction, so, sometimes, for quick virus detection, total RNA extraction is preferred. 

**Figure 2:**
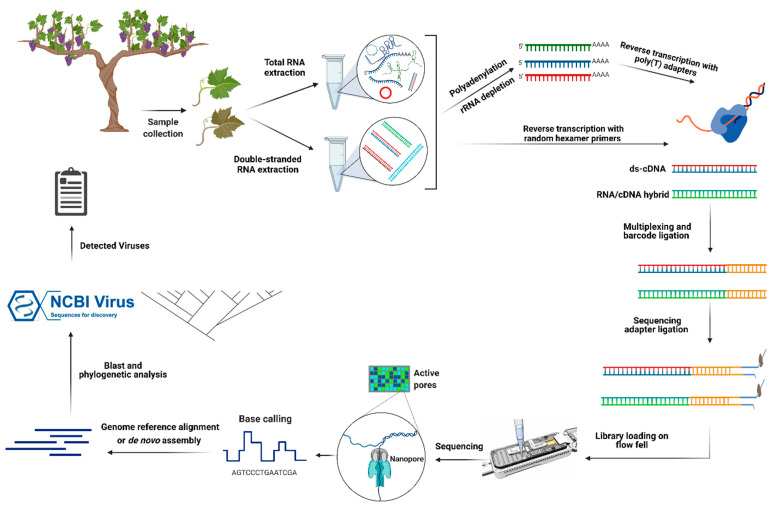
Illustration of the principles of nanopore direct cDNA and RNA sequencing for grapevine RNA virus detection. In direct RNA sequencing, after total RNA extraction, rRNAs are depleted using specific probes. To capture non-poly(A) viral RNAs, polyadenylation is performed. The first strand of cDNA is transcribed by using poly(T) adapters. In addition, different barcodes can be used to multiplex several libraries and sequence them using one flow cell. Then, the sequencing adapter is ligated, and the library (or pooled libraries) is loaded onto a flow cell. Finally, the sequencing step is run by setting up different options in MinKNOW software. After basecalling, downstream analysis steps are performed, and the list of detected viruses is extracted. In direct cDNA sequencing, after dsRNA extraction, double-stranded cDNAs are synthesized with random hexamer primers, and the subsequent steps are similar to direct RNA sequencing. Created with BioRender.com (accessed 15 September 2021).

Eighteen different reagent kits for the nanopore sequencing of various genetic materials have been commercialized, and four of them were used in different plant virus detection projects (Table 2). The direct cDNA sequencing kit with multiplexing capability is an option to sequence cDNA synthesized from purified dsRNA or total RNAs. Although cDNA synthesis in this protocol is based on the strand-switching procedure and oligo dT-containing VN primers (V for dA, dC, or dG and N for dA, dC, dG, or dT), cDNA can also be synthesized using random hexamer primers to target both poly(A) and non-poly(A) viral RNAs (Figure 2). The latter is an important element to consider, as many RNA viruses do not produce poly(A) tailed RNAs. Through poly(A) tailing, the enrichment of poly(A) and non-poly(A) viral RNAs is also possible, but cDNA synthesis based on the strand-switching mechanism VN primers can produce internal priming and template-switching artifacts. In the case of internal priming, the initiation of transcription is not from the poly(A) tail, and the poly(T) primer anneals to an adenine-rich region (six or more consecutive adenines, or 12 adenines out of 20 nucleotides) of the transcript, and an internal priming artifact is elongated. With template-switching artifacts, the DNA polymerase dislocates during the elongation step and initiates synthesis at a homologous sequence (sometimes as short as three adenines) on another template [47]. Such artifacts in RNA virus detection by nanopore sequencing have been reported [48]. To overcome these problems, cDNA synthesis using random hexamers can be an appropriate alternative procedure [49]. 

Another nanopore sequencing kit that has recently attracted more attention is the direct RNA sequencing kit. The association of motor protein, as the regulator of the nucleic acid molecule translocation, with the RNA molecule indicates the low translocation velocity of RNA through the nanopore protein, which means that the passing speed of RNA within the nanopore is slow enough to be measured and sequenced. Consequently, using algorithms adapted to this platform, current intensity fluctuations induced by the passage of RNA molecules through the nanopore can be converted into sequence information [50,51].

In contrast to the direct cDNA sequencing kit, the cDNA synthesis step (the first strand of cDNA) is optional for direct RNA sequencing. After the ligation of a double-stranded RT Adapter (RTA) and RNA sequencing adapter (RMX), native RNA can be sequenced directly [51]. Nevertheless, first-strand cDNA synthesis is recommended to resolve the structural complexity of RNA molecules and prevent the production of different-sized fragment reads during sequencing. When an RNA has a complex structure, the nanopore protein may be blocked, and the sequencing will stall [52]. Additional obstacles encountered with direct RNA sequencing compared to direct cDNA sequencing include lower throughput [53], higher sequencing error rate [54], and a lack of commercial barcodes for multiplexing. However, a new method, DeePlexiCon, has recently been described for barcoding and demultiplexing direct RNA sequencing libraries [51]. 

As a common limitation among all nanopore sequencing kits, high sequencing error rates limit the accurate characterization of quasi-species, new strains or subtypes of a virus, and the de novo assembly of viral reads [42,55,56]. In a given infected plant, closely related viral strains can be present with high average nucleotide identity (NI), and the assembly of individual strains present in low abundance or with low variation is complicated and challenging [57,58]. Even so, the capability of the direct RNA sequencing kit for identifying viral strains with 20% to 40% divergence in terms of NI has been demonstrated [59]. In addition, Haploflow, a new strain-resolving assembler, has been described, which considers the differential coverage between strains to deconvolute the assembly graph into strain-resolved genome assemblies and has been used to reconstruct viral strain genomes from human cytomegalovirus-positive samples and SARS-CoV-2 wastewater samples [57]. Moreover, using the full reference sequence of the virus and a BLAST search with phylogeny is recommended for viral phylogeny and viral variation studies [56]. For studying genetic recombination, adaptive evolution, or resistance-breaking mutations in plant virology, nanopore sequencing is less than ideal until improvements in error rates are developed [42]. However, this technology is still an interesting option for virus detection from a variety of biological samples. 

## 5. Detection of Grapevine DNA Viruses by Nanopore Ligation and Rapid Sequencing 

Since several single-stranded DNA viruses, such as GRBV, and double-stranded DNA viruses, such as GVCV, can infect grapevines, two library preparation kits, ligation and rapid sequencing, are available for detecting them. Indeed, similar viruses, such as *Cowpea bright yellow mosaic virus* (CoBYMV) and *African cassava mosaic virus* (ACMV), have been detected using ligation and rapid sequencing kits, respectively [36,38]. Although both sequencing kits can be used for detecting DNA viruses, the throughput of rapid sequencing is lower (25% lower) than that of ligation sequencing. However, the library preparation time for rapid sequencing is much shorter (~10 minutes) than that of ligation sequencing (~60 minutes), and the amount of input material necessary for this kit is lower (400 ng DNA) than that of the ligation sequencing kit (1000 ng DNA) [60]. 

## 6. Adaptive Nanopore Sequencing for Real-Time Virus Detection

For the real-time detection of known viruses, adaptive sequencing by nanopore sequencing devices is possible. In this kind of sequencing, through partial mapping reads against desirable reference genomes, undesired reads can be ejected from the nanopore through provisionally reversing the voltage, and only targeted sequences are sequenced [61,62]. Adaptive sequencing can be done by mapping raw signals [62] or basecalled data against reference genomes [63]. Sufficient computing power (especially GPU power) is needed for basecalling. Therefore, raw signal mapping tools, such as Sigmap [62], can be used for fast and on-site virus detection. This kind of sequencing reduces sequencing costs and laborious library preparation for detecting pathogens such as viruses quickly.

## 7. Viroids and Virusoids 

There are various infectious long non-coding RNAs that originated from viruses or viroids. Viroids are single-stranded circular non-coding RNAs that are the smallest infectious entities (between 246 and 401 nt in length) of plants and do not encode any protein. Viroids can replicate individually in the host plant nucleus or chloroplast by using host RNA polymerases through asymmetric and symmetric rolling-circle mechanisms [64]. Although many viroids were initially identified by their symptoms on infected plants, it is now known that viroids can also infect hosts asymptomatically [65]. As with viruses, viroid-derived small RNAs (vd-sRNAs) accumulate in infected plant tissues and can be used to detect viroids [66]. 

As minor mutations in viroid sequences can dramatically affect the viroid movement [67], sequencing the full length of viroids will help us to better understand their functionality and pathogenicity in the grapevine. Thus far, seven different viroid species from the *Pospiviroidae* family, such as *Hop stunt viroid*, *Australian grapevine viroid*, *Grapevine latent viroid*, and *Grapevine yellow speckle viroid-1,2,3* and a tentative and an unclassified viroid, Grapevine hammerhead viroid-like RNA, have been identified in grapevines [68,69]. Grapevine viroids do not induce severe symptoms in grapevines singly unless in particular environmental conditions. They can cause severe symptoms in other host plants and be the primary disease agents. They seem to play a role in some viral diseases through coinfection with other grapevine viruses [65,70]. Therefore, molecular, biological, and epidemiological analysis of grapevine viroids and viroid-like RNAs is needed. 

As with viroids, virusoids (or small circular satellite RNAs) are other circular, non-coding RNAs ranging from 220 to 457 nt in length. Virusoids replicate through a rolling-circle mechanism based on RNA intermediates and are dependent on helper viruses or host plants. In contrast to viroids, virusoids are encapsidated by the helper virus coat protein [64,71]. The similarities between viroids and virusoids have led to the hypothesis that these circular RNAs may have a monophyletic origin [71]. Several contradictory biological functions have been attributed to virusoids; for example, a virusoid decreased the accumulation of *Tobacco ringspot virus* (TRSV) as a helper virus and attenuated the symptoms in infected tobacco samples [72]. In contrast, some virusoids produce small RNAs (21–24 nt) similar to microRNAs and small interfering RNAs, downregulate some host mRNAs, and are involved in pathogenesis. Helper viruses of identified virusoids are from genera *Sobemovirus*, *Nepovirus*, and *Polerovirus* [71]. Since viroids and virusoids are circular, their identification and detection by nanopore sequencing is similar to circRNA detection, which is described in the following section. 

## 8. Identification of Long Non-Coding RNAs (lncRNAs) and Circular RNAs (circRNAs) 

Recently, the function of long non-coding RNAs (lncRNA) and circular RNA (circRNA) as new RNA regulators in plant developmental processes and stress responses, especially in plant–virus interactions, has been studied [73]. Long non-coding RNAs are transcribed by RNA polymerase II, III, IV, and V [74,75,76] and are longer than 200 nt [77]. There are five main categories of lncRNAs: intronic, intergenic, sense, bidirectional, and natural antisense lncRNAs [78]. Thus far, many lncRNAs have been identified in different plants, such as watermelon [73], maize [74], Arabidopsis [79,80], rice, tomato [81,82], melon [83], cucumber [84], and Chinese cabbage [85]. However, there are limited studies about the function of lncRNAs in plant–virus interactions [73,80,82,86]. 

Circular RNAs (circRNAs), as single-stranded and stress-inducible RNAs, are generated by rare back-splicing events from precursor mRNAs (Pre-mRNAs). In a back-splicing event, an upstream 3′ splice site is ligated to a downstream 5′ splice site through a 3′–5′ phosphodiester bond [87,88] CircRNAs can originate from exonic, intronic, and intergenic regions of genomes [89,90]. Their expression depends on tissue and cell type, developmental stages, and biotic or abiotic stresses [83,91,92,93]. Although, until now, 142,115 cricRNAs in 20 plant species have been reported and deposited in PlantcircBase [94], the functionality of plant circRNAs is still unknown and understudied. Several recent studies have indicated that circRNAs could be miRNA sponges or competing endogenous RNAs and regulate the expression of paternal genes through alternative splicing events [95,96]. In grapevine, from 10 developmental stages of leaf, inflorescence, and berry tissues, 56,441 lncRNAs were identified that regulated developmental transitions and were involved in biosynthetic and secondary metabolic pathways, photosynthesis, and oxidative phosphorylation [97]. In addition, by using different circRNA prediction algorithms, grapevine circRNA biogenesis was investigated, and 475 differentially expressed circRNAs during cold stress in grapevine leaves were identified [83]. However, less attention has been paid to the potential function of circRNAs in plant–virus interactions [73,98]. Since lncRNAs and circRNAs are involved in different biological processes and have specific expression patterns under biotic and abiotic stresses, their potential regulatory roles at the transcriptional and post-transcriptional levels during virus infection should be investigated further. Such investigation will open a new window into grapevine virology and disease management. 

**Figure 3:**
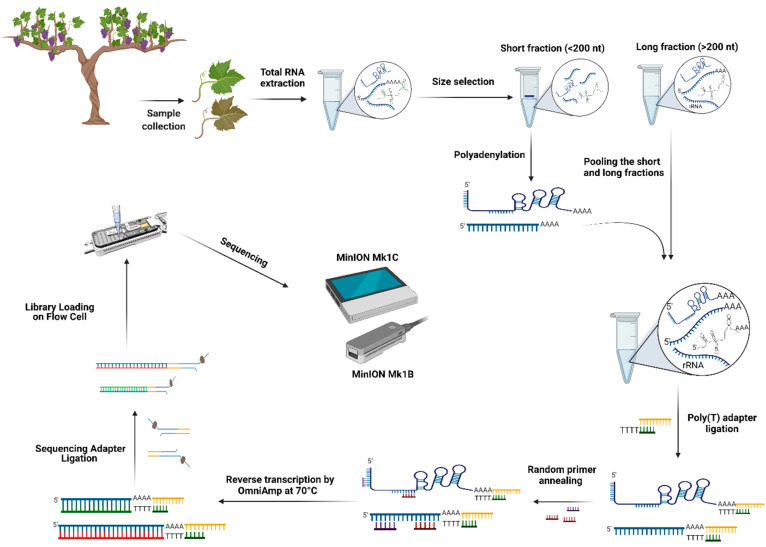
Non-coding RNA and poly(A) tail RNA sequencing in infected grapevine samples by nanopore direct RNA sequencing. Created with BioRender.com (accessed 15 September 2021).

Although many lncRNAs and circRNAs have been identified by the advent of short-read sequencing [99], the prediction of different isoforms and splice events cannot be achieved accurately by short-read sequencing [100]. In addition, intergenic and intronic regions that originated from insertions or remnants of transposable elements (TEs) are the sources of various lncRNAs, and the association of lncRNAs with these kinds of repetitive sequences, such as TEs, has been suggested [101,102]. Until now, many bioinformatics pipelines have ignored ambiguously mapped reads in lncRNA identification, and the annotation of many lncRNAs is made challenging by the use of short-read sequencing [101,103]. Recently, long-read sequencing has allowed the sequencing of full-length RNA molecules and identification of 796 triticale lncRNAs and hundreds of unknown genic and intergenic lncRNAs. It was also possible to determine the association of these lncRNAs with the remnants of Class I and Class II transposable elements [101]. However, in this study, the presence of non-poly(A) lncRNAs was not considered. Alternatively, Saville et al. (2021) reported that a modified nanopore sequencing protocol, NERD-seq, can capture and sequence many poly(A) and non-polyadenylated RNAs and non-coding RNAs. This technique allows the comprehensive study of the epitranscriptome by using nanopore direct RNA sequencing. Briefly, in this protocol, total extracted RNAs were split into two different-sized fractions, long and short RNA fractions, by a column-based size enrichment approach. Poly(A) tailing was performed on the short-sized fraction to keep the non-polyadenylated RNAs in the sequencing library; however, the poly(A) tailing process improves the accuracy of short-read sequencing by nanopore sequencing. In long-sized fractions, rRNA depletion was performed, and then two fractions were pooled together [104]. One of the main problems in nanopore direct RNA sequencing is the inhibition of the sequencing process by highly structured RNA regions [104,105], which may inhibit the pulling of the RNA molecule through the nanopore. An alternative solution is first-strand cDNA synthesis through reverse transcription. Although the first-strand cDNA is not sequenced, it improves the stability of the RNA strand during the sequencing step and resolves highly structured RNA regions in non-coding RNAs [106]. Based on the nanopore direct RNA sequencing manufacturer’s protocol, the reverse transcription is done at between 45 °C and 50 °C, and some highly structured non-coding RNAs are not transcribed at this temperature [104]. Therefore, the reverse transcription process is interrupted, and cDNAs of different lengths are produced. In the NERD-seq procedure, two-step reverse transcription is used to solve this problem. This procedure simplifies the RNA unfolding through OmniAmp, a loop-mediated isothermal amplification polymerase with random primers for initial reverse transcription at 70 °C to produce the small first-strand cDNA fragments. After this, these small cDNA fragments are displaced by the cDNA strand of poly(T) reverse transcription [104]. This methodology offers the opportunity to characterize many kinds of non-coding RNAs involved in grapevine–virus interactions and to investigate the regulatory gene networks affected by viral infection (Figure 3). 

**Figure 4:**
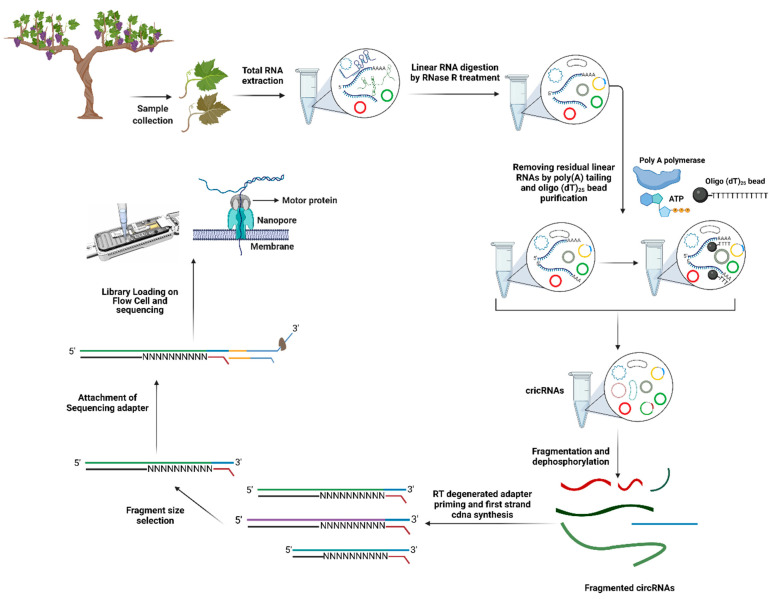
Detection of circular RNAs, viroids, and virusoids in infected grapevine samples by nanopore direct RNA sequencing. Created with BioRender.com (accessed September 15th 2021).

Concurrent with the advancement in next-generation sequencing, many circRNAs in plants have been characterized by various algorithms and bioinformatics tools, such as CIRI [107], KNIFE [108], and CIRCexplorer [109]. Most circRNA identification tools are based on short read alignment. A large number of circRNAs originate from exonic regions, which makes distinguishing circRNAs from overlapping regions of their pre-mRNAs challenging [110,111]. Hence, long-read sequencing by nanopore can be used for plant circRNA characterization and exploited to identify infectious circular RNAs, such as viroids and virusoids from infected grapevine samples (Figure 4). 

Two interesting library preparation methodologies for circRNA sequencing based on nanopore direct RNA and cDNA sequencing are introduced in the following section. A novel library preparation procedure was reported in a recent study to characterize 470 unique circRNAs and their N6-methyladenosine (m^6^A) modifications in moso bamboo (*Phyllostachys edulis*) by using nanopore direct RNA sequencing. Concisely, extracted total RNAs were treated with RNase R to remove linear RNAs. Since the digestion of linear RNAs was not entirely achieved by RNase R treatment, poly(A) tailing was performed to deplete the remaining linear RNAs through oligo (dT)_25_ beads. In the next step, rRNAs were depleted, and then the purified circRNAs were fragmented and dephosphorylated. Finally, after first-strand cDNA synthesis using a customized RT adapter containing degenerate primers, the sequencing adapter was ligated. Although this library preparation approach successfully identified circRNAs and their m^6^A modifications, the low sequencing depth of nanopore direct RNA sequencing remained the main obstacle for quantitative analysis [112]. Moreover, the fragmentation step in this protocol produces different sizes of fragments (especially short reads), which reduces pore viability, motor protein fuel reserves, and overall yield [104]. Consequently, higher enrichment of circRNAs can be achieved through fragment size selection. In one study, the portion of the full length of circRNAs in a library without a size selection step was 1%, while in medium and long selected-fragment libraries, three-fold and six-fold more full-length circRNAs were captured, respectively [111].

In contrast to the above library preparation protocol, a recent study showed the application of nanopore cDNA sequencing for identifying various kinds of circRNAs in mouse brain samples [111]. In this protocol, the chemical fragmentation of circRNAs was omitted, and ds-cDNAs of circular RNAs were sequenced. After total RNA extraction, rRNA depletion, poly(A) tailing, and digestion of linear RNAs were performed. After reverse transcription with random primers and cDNA amplification, fragment size (400 bp, 600 bp, and 1000 bp) selection with magnetic beads were followed. Finally, library preparation and sequencing adapter ligation were done sequentially. The library was primed on a flow cell (R9) and sequenced using the MinION platform. In this study, the library preparation protocols were suitable for identifying different known types of circRNAs in mouse brain samples. A novel algorithm, CIRI-long, was used to construct a new type of intronic self-ligated circRNA [111].

## 9. Detection of Viral RNA Modifications by Nanopore Sequencing

Recently, a novel area in virology, viral RNA epitranscriptomics, has been investigated with the advent of new RNA modification site mapping techniques, such as nanopore direct RNA sequencing. Many questions about the functionality of these RNA modifications have been raised. RNA modifications appear through adding biochemical groups, such as a methyl group, to adenosine, cytosine, uracil, and guanosine nucleotides [113]. Although many studies have been done for mapping the various RNA modifications, such as N6-methyladenosine (m^6^A), 5-methylcytidine (m^5^C), or N4-acetylcytidine (ac^4^C) on eukaryote mRNAs [113,114], viral RNA modifications have received little investigation. However, RNA modifications of viral RNAs do appear to be important. For instance, m^6^A on HIV-1 mRNA can modulate HIV-1 gene expression and affects the HIV-1 RNA stability and exportation of its RNA from nucleus [115]. In addition, m^6^A not only can decrease virus pathogenicity in Influenza virus but also promotes infection kinetics [115,116]. Likewise, ablation of an RNA demethylase in Arabidopsis resulted in increased m^6^A methylation of alfalfa mosaic virus RNA. This correlated with reduced systemic spread of the virus, suggesting that viral RNA methylation dynamics may play an important role in virus infections in plants [117]. Until now, nine RNA modification types on RNA or DNA human viruses have been mapped through antibody, protein CLIP (Class II-associated invariant chain peptide), biological, and nanopore mapping procedures. For instance, nanopore direct RNA sequencing technology was used to identify m^5^C methylation sites on the human coronavirus genome through the bioinformatics tool called Tombo [118]. Moreover, several bioinformatics tools, such as EpiNano, MINES, Nanocompore, and ELIGOs, have been developed for profiling m^6^A modification sites on different types of RNAs by using nanopore sequencing data [119,120]. In addition to the identification of RNA modifications on viral RNAs, several functions in biological events, such as viral RNA trafficking, the degradation of viral RNA, the splicing of viral RNA, and immune evasion by viral RNA, have been proposed [113]. The continuous improvement of nanopore sequencing in the near future will allow researchers to answer many questions about biological processes in plant virology and help us to understand how RNA modifications of viral genomes can influence their host as well as their new variants or other viruses. 

## 10. Perspectives

Through the advent of nanopore sequencing, a new way to sequence and quantify RNA and DNA has been established. Soon, nanopore technology will have a tremendous impact on plant virus detection, RNA modification research, plant epigenomics, epitranscriptomics, and other aspects of plant biology and virology. The sequencing error rate of this technology initially was significantly higher than other high-throughput sequencers. However, the technology is being continuously improved to increase its accuracy by optimizing new nanopore proteins and motor proteins, designing new basecalling algorithms, changing the chemistry of library preparation kits, and testing a new sequencing orientation (Outy). This technology is a cost- and time-effective method for addressing various aspects of grapevine virology, such as massively parallel sequencing (MPS), grapevine virus detection, virus–plant interactions, and viral RNA modifications. Although a number of challenges, such as error correction and base modification detection, need to be resolved, the valuable potential of nanopore technology in grapevine virology should not be overlooked.

## Figures and Tables

**Table 1:** General properties of commercial nanopore sequencer machines and their flow cells.

Sequencer	Flow Cell	Maximum Yield per Flow Cell (Gb)	Flow Cell Number in Each Running	Maximum Running Time (Hours)	Nanopore Channel Number in Each Flow Cell
MinION Mk1B/Mk1C	MinION (R9.4.1 or R10.3) /Flongle	50 for MinION/2.8 for Flongle	1	72 for MinION/16 for Flongle	512 for MinION/126 for Flongle
GridION	MinION (R9.4.1 or R10.3)/Flongle	50 for MinION/2.8 for Flongle	5	72 for MinION/16 for Flongle	512 for MinION/126 for Flongle
PromethION 24/48	PromethION	290	24/48	72	2675

**Table 2:** List of plant viruses detected using nanopore sequencing technology.

Virus Name	Host Plant	Nucleic Acid Extraction	Library Preparation Kit	References
*Sowthistle yellow vein virus* (SYVV)	*Sonchus oleraceus* L.	Total RNA	Direct RNA Sequencing	[37]
*Cowpea bright yellow mosaic virus* (CoBYMV)	*Vigna unguiculata*	Total DNA	Ligation Sequencing	[38]
*Plum pox virus*	*Prunus persica*	Total RNA	Ligation Sequencing	[39]
*Wheat streak mosaic virus* (WSMV) *Triticum mosaic virus* (TriMV) *Barley yellow dwarf virus* (BYDV)	Wheat	Total RNA	Ligation Sequencing	[40]
*Tomato yellow leaf curl virus* (TYLCV) *Watermelon chlorotic stunt virus* (WmCSV) *Tomato brown rugose fruit virus* (ToBRFV) *Cucumber green mottle mosaic virus* (CGMMV) Zucchini yellow mosaic virus (ZYMV)	*Solanum lycopersicum* *Cucumis sativus* *Citrullus lanatus* *Cucurbita moschata*	Total RNA Total DNA	Direct RNA Sequencing Ligation Sequencing	[41]
*Dioscorea bacilliform viruses* (DBVs) *Yam mild mosaic virus* (YMMV) *Yam chlorotic necrosis virus* (YCNV)	*Dioscorea alata*	Total RNA	PCR-cDNA Sequencing Kit	[42]
*Potato virus Y* (PVY) *Potato virus X* (PVX) *Potato virus S* (PVS) *Potato leafroll virus* (PLRV)	*Solanum tuberosum* L.	Total RNA	Ligation Sequencing Kit	[43]
*East African cassava mosaic virus* (EACMV) *African cassava mosaic virus* (ACMV) *Tobacco leaf curl virus* (TLCV)	*Manihot esculenta*	Total DNA	Rapid Barcoding Kit	[36]

## Data Availability

Not applicable.

## Supplementary Materials

The following are available online at https://www.mdpi.com/article/10.3390/plants10112355/s1, Table S1: List of viruses identified in grapevine (updated from Fuchs 2020).
