# Supplementary material for: Grapevine Virology in the Third-Generation Sequencing Era: From Virus Detection to Viral Epitranscriptomics
Source: Plants (Basel). 2021 Oct 31;10(11):2355. doi: 10.3390/plants10112355 (PMC8623739; doi:10.3390/plants10112355)
Supplement: Supplementary file 1 [file plants-10-02355-s001.zip › plants-1404750-SI.pdf]

Table S1. List of viruses identified in grapevine (updated from Fuchs 2020).

Genome	Family	Genus	Species	Virion shape	Disease
**(+)ssRNA**	*Alphaflexiviridae*	*Potexvirus*	*Potato virus X* (PVX)	Filamentous	None
	Betaflexiviridae	*Fivirus*	*Grapevine Kizil Sapak virus* (GKS)	Filamentous	Unknown
		Foveavirus	*Grapevine rupestris stem pitting-associated virus* (GRSPaV)<br>*Grapevine virus T* (GVT)	Filamentous	Rugose wood<br>Unknown
		Trichovirus	*Grapevine berry inner necrosis virus* (GINV)<br>*Grapevine Pinot gris virus* (GPGV)	Filamentous	Berry inner necrosis<br>leaf deformation and mottling
		Vitivirus	*Grapevine virus A* (GVA)<br>*Grapevine virus B* (GVB)<br>*Grapevine virus D* (GVD)<br>*Grapevine virus E* (GVE)<br>*Grapevine virus F* (GVF)<br>*Grapevine virus G* (GVG)<br>*Grapevine virus H* (GVH)<br>Grapevine virus I (GVI)<br>*Grapevine virus J* (GVJ)<br>*Grapevine virus K* (GVK)<br>*Grapevine virus L* (GVL)<br>*Grapevine virus M* (GVM)	Flexuous filamentous	Rugose wood<br><br>Unknown<br>Unknown<br>Rugose wood
	Bromoviridae	*Alfamovirus*	*Alfalfa mosaic virus* (AMV)	Bacilliform	Yellow mosaic
		Anulavirus	*Amazon lily mild mottle virus* (ALiMMV)<br>*Grapevine line pattern virus* (GLPV)	Isometric	None<br>Line pattern
		Cucumovirus	*Cucumber mosaic virus* (CMV)	Isometric	Unknown
		Ilarvirus	*Grapevine angular mosaic virus* (GaMoV)<br>*Grapevine virus S* (GVS)	Isometric<br>Unknown	Angular mosaic<br>Unknown
	Closteroviridae	*Closterovirus*	*Grapevine leafroll-associated virus 2* (GLRaV2)	Filamentous	Leafroll/ Incompatibility

Genome	Family	Genus	Species	Virion shape	Disease
		Ampelovirus	*Grapevine leafroll-associated virus 1* (GLRaV1)<br>*Grapevine leafroll-associated virus 3* (GLRaV3)<br>*Grapevine leafroll-associated virus 4* (GLRaV4)<br>*Grapevine leafroll-associated virus 13* (GLRaV13)	Filamentous	Leafroll
		Velarivirus	*Grapevine leafroll-associated virus 7* (GLRaV7)	Filamentous	Leafroll
	Luteoviridae	*Enamovirus*	*Grapevine enamovirus 1* (GEV1)	Isometric	None
		Unassigned	*Grapevine Ajinashika virus* (GAgV)	Isometric	Unknown
	Partitiviridae	*Deltapartitivirus*	*Grapevine cryptic virus* (GCV)	Unknown	Unknown
	Potyviridae	*Potyvirus*	*Bean common mosaic virus* (BCMV)	Filamentous	Unknown
	Secoviridae	*Cheravirus*	*Apple latent spherical virus* (ALSV)	Isometric	Unknown
		Fabavirus	*Broad bean wilt virus* (BBMV)<br>*Grapevine fabavirus* (GFabV)	Isometric	Unknown<br>Shine Muscat showing disease
		Nepovirus	*Artichoke Italian latent virus* (AILV)<br>*Arabis mosaic virus* (ArMV)<br>*Blueberry leaf mottle virus* (BBLMV)<br>*Cherry leafroll virus* (CLRV)<br>*Grapevine Anatolian ringspot virus* (GARSV)<br>*Grapevine Bulgarian latent virus* (GBLV)<br>*Grapevine deformation virus* (GDeV)<br>*Grapevine chrome mosaic virus* (GCMV)<br>*Grapevine fanleaf virus* (GFLV)<br>*Grapevine Tunisian ringspot virus* (GTRV)<br>*Peach rosette mosaic virus* (PRSM)<br>*Raspberry ringspot virus* (RpRSV)<br>*Tobacco ringspot virus* (TRSV)<br>*Tomato ringspot virus* (ToRSV)<br>*Tomato black ring virus (TBRV)*	Isometric	Leaf degeneration
		Unassigned	*Strawberry latent ringspot virus* (SLRSV)	Isometric	Leaf degeneration
	Tombusviridae	*Carmovirus*	*Carnation mottle virus* (CarMV)	Isometric	Roditis leaf discoloration
		Necrovirus	*Tobacco necrosis virus D* (TNV-D)	Isometric	Unknown

Genome	Family	Genus	Species	Virion shape	Disease
		Tombusvirus	*Grapevine Algerian latent virus* (GALV)	Isometric	Unknown
			Petunia asteroid mosaic virus (PAMV)		
	Tymoviridae	*Marafivirus*	*Blackberry virus S* (BlVS)	Isometric	Unknown
			Grapevine asteroid mosaic-associated virus (GAMaV)		Asteroid mosaic
			Grapevine Syrah virus 1 (GSyV1)		Unknown
		Maculavirus	*Grapevine fleck virus* (GFkV)	Isometric	Fleck
			Grapevine red globe virus (GRGV)		Unknown
		Gratylivirus	*Grapevine-associated tymo-like virus* (GaTLV)	Isometric	Unknown
	Virgaviridae	*Tobamovirus*	*Grapevine virga-like virus* (GVLV)	Rod	Unknown
			Tobacco mosaic virus (TMV)		
			Tomato mosaic virus (ToMV)		
	Unassigned	*Idaeovirus*	*Raspberry bushy dwarf virus* (RBDV)	Isometric	Unknown
		Sobemovirus	*Sowbane mosaic virus* (SoMV)	Isometric	Unknown
		Virtovirus	*Grapevine virus satellite (GV-Sat)*	Isometric	Unknown
		Unassigned	*Grapevine labile rod-shaped virus (GLRSV)*	Rod	Unknown
			Grapevine stunt virus (GSV)	Isometric	
**(−)ssRNA**	*Bunyaviridae*	*Tospovirus*	*Tomato spotted wilt virus* (TSWV)	Isometric	Unknown
	Phenuiviridae	*Rubodvirus*	*Grapevine Garan dmak virus* (GGDV)	Isometric	Unknown
			Grapevine Muscat rose virus (GMRV)		
**dsRNA**	*Endornaviridae*	*Endornavirus*	*Grapevine endophyte endornavirus* (GEEV)	Does not have any gene for capsid protein, no virion	None
	Reoviridae	Oryzavirus (?)	*Grapevine Cabernet Sauvignon reovirus* (GCSV)	Isometric	Unknown
**ssDNA**	*Geminiviridae*	*Begomovirus*	*Grapevine begomovirus A* (GBVA)	Twinned	Unknown
		Grablovirus	*Grapevine red blotch virus* (GRBV)	Twinned	Red blotch
			Wild Vitis latent virus 1 (WVV1)		
		Unassigned	*Grapevine geminivirus A* (GGVA)	Twinned	Unknown
			Temperate fruit-decay-associated virus (TFDaV)		
**dsDNA**	*Caulimoviridae*	*Badnavirus*	*Grapevine vein clearing virus* (GVCV)	Bacilliform	Vein clearing
			Grapevine badnavirus 1 (GBV1)		Unknown
			Grapevine Roditis leaf discoloration-associated virus (GRLDaV)		Roditis discoloration

1. Fuchs, M. Grapevine viruses: A multitude of diverse species with simple but overall poorly adopted management solutions in the vineyard. *Journal of Plant Pathology* **2020**, *102*, 643-653.
